# Clinical Predictors of the Rapid Progression and Revascularization of Coronary Non-Target Lesions: A Serial Angiographic Study

**DOI:** 10.31083/j.rcm2507251

**Published:** 2024-07-08

**Authors:** Wei Wang, Haobo Xu, Jiansong Yuan, Chao Guo, Fenghuan Hu, Weixian Yang, Xiaoliang Luo, Rong Liu, Shengwen Liu, Jilin Chen, Shubin Qiao, Jingang Cui, Juan Wang

**Affiliations:** ^1^Department of Cardiology, Fuwai Hospital, National Center for Cardiovascular Diseases, Chinese Academy of Medical Sciences, 100037 Beijing, China

**Keywords:** coronary heart disease, non-target lesion, rapid progression, revascularization, predictor

## Abstract

**Background:**

Rapid progression of coronary non-target lesions is essential for the determination of future cardiovascular events. Clinical factors that predict rapid progression of non-target lesions are unclear. The purpose of this study was to identify the clinical predictors of rapid progression and revascularization of coronary non-target lesions.

**Methods:**

Consecutive patients with coronary heart disease who had undergone two serial coronary angiograms were enrolled. All coronary non-target lesions were identified and evaluated at both procedures. Multivariable Cox regression analysis was used to investigate the clinical risk factors associated with rapid progression or revascularization of coronary non-target lesions.

**Results:**

A total of 1255 patients and 1670 lesions were enrolled. In this cohort of patients, 239 (19%) had rapid progression and 186 (14.8%) underwent revascularization. At the lesion level, 251 (15.0%) had rapid progression and 194 (11.6%) underwent revascularization. The incidence of lesion revascularization and myocardial infarction was significantly higher in patients with rapid progression. In multivariable analyses, hypertension (hazard ratio [HR], 0.76; 95% confidence interval [95% CI], 0.58–1.00; *p* = 0.049), ST-segment elevation myocardial infarction (STEMI) (HR, 1.46; 95% CI, 1.03–2.07; *p* = 0.035), glycosylated hemoglobin (HR, 1.16; 95% CI, 1.01–1.33; *p* = 0.039) and lesion classification (B2/C versus A/B1) (HR, 1.73; 95% CI, 1.27–2.35; *p* = 0.001) were significant factors associated with rapid progression. The level of triglycerides (HR, 1.10; 95% CI, 1.00–1.20; *p* = 0.040) and lesion classification (B2/C versus A/B1) (HR, 1.53; 95% CI, 1.09–2.14; *p* = 0.014) were predictors of lesion revascularization.

**Conclusions:**

Hypertension, STEMI, glycosylated hemoglobin and lesion classification may be used as predictors of rapid progression of coronary non-target lesions. The level of triglyceride and lesion classification may predict the revascularization of non-target lesions. In order to prevent future cardiovascular events, increased attention should be paid to patients with these factors.

## 1. Introduction

Coronary heart disease (CHD) is one of the leading causes of death in both developed and developing countries [1]. Although coronary atherosclerosis is believed to be a chronic process that would progress over many years, it has been increasingly noted to progresses over a few months to 1–2 years in patients with accelerated atherosclerosis [2, 3, 4]. Serial studies involving angiographic data have demonstrated that coronary lesions with mild or moderate stenosis become progressively more stenosed before the acute event occurs [5]. Yokoya *et al*. [6] and Kaski *et al*. [7] showed that acute coronary syndromes are manifested in 50–70% patients who have rapid progression of coronary lesions. Therefore, identifying clinical predictors of the rapid progression of coronary lesions is of great importance.

Results from previous studies have shown that risk factors such as cigarette smoking and high cholesterol levels contribute to rapid progression of mild to moderate coronary stenoses [8, 9]. However, these studies were mainly conducted before 2010 and the control of these risk factors was not as strict as it is today. In addition, patients who received strict risk factor control still had rapid progression of coronary lesions [10, 11].

The present study aims to investigate clinical predictors of the rapid progression and revascularization of coronary non-target lesions based on two serial coronary angiographies (CAGs) in order to provide strategies to modify this accelerated process.

## 2. Materials and Methods

### 2.1 Study Population

Consecutive patients with CHD who had undergone two serial coronary angiograms from January 2010 to September 2014 were retrospectively enrolled in our hospital, including those with acute coronary syndrome or stable angina. Patients were excluded as follows: renal dysfunction, history of coronary artery bypass graft surgery, active malignant tumor, valvular heart disease with clinically significance, severe conduction disturbances and significant arrhythmias.

In total, 1255 patients were eventually enrolled in the present study. During the initial CAG, the decision to perform percutaneous coronary intervention (PCI) was left to the discretion of the operator. The second CAG was prompted by clinical presentation, an abnormal stress test demonstrating myocardial ischemia, or a routine follow-up angiogram. The serial CAGs were all conducted within a two-year period in accordance with the findings of previous studies [6, 7, 9].

A coronary non-target lesion refers to a de novo stenotic lesion that does not contribute to ischemic presentation, or yield positive results in functional ischemic testing, as previously defined [12, 13]. All coronary non-target lesions were identified during the initial CAG. They were measured by quantitative coronary angiography (QCA) during both procedures. The outcomes of the second CAG were recorded, encompassing rapid progression, revascularization of coronary non-target lesions, and myocardial infarction.

All patients in the study were treated with standard medical treatment between the two CAGs. Data including demographics, medical history, biochemical data and detailed coronary angiography were collected. The study was conducted in accordance with the principles of the Declaration of Helsinki and had the approval of the Institutional Review Board of Fuwai Hospital (No. 2005-1516). All participants provided written informed consent in the present study.

### 2.2 Process of CAG and QCA Measurement

The selective CAG was performed after administrating an intracoronary injection of glyceryl nitrate. QCA data from both CAG procedures were compared to assess the progression of angiographic lesions. The same projection was used for each pair of coronary angiograms. Coronary angiograms were reviewed by two independent observers experienced in interpreting angiograms who were blinded to the clinical data. The analysis of QCA involved using the Judkins coronary catheter shaft for calibration to obtain absolute measurements in millimeters. The distortion of radiographic pincushion has been corrected. Measurements were taken on end-diastolic frames for each segment, where the vessel appeared to be maximally stenosed. Measurement of the lesion length, reference diameter, minimum lumen diameter and the percentage of diameter stenosis were performed.

### 2.3 Rapid Progression and Revascularization of Coronary Non-Target Lesion

The primary groups included the rapid progression and non-progression of the non-target lesion. The rapid progression was interpreted as an increase in the percentage of diameter stenosis, which was calculated by subtracting the diameter stenosis at the second CAG from the diameter stenosis at the first CAG. The definition of rapid progression has been previously described [13]. A coronary non-target lesion is deemed to have rapid progression with the presence of any of the following: a reduction in diameter of at least 10% required from a pre-existing stenosis that was at least 30% or a reduction in diameter of at least 30% required from a pre-existing stenosis that was less than 30%, or progression to total occlusion at the second CAG. Patients were grouped into the progression group if they had at least one rapidly progressing lesion. Patients who had no rapid progression were grouped into the non-progression group.

The secondary groups involved revascularization and non-revascularization of the non-target lesion. Revascularization of the non-target lesions at the second CAG was performed based on ischemic symptoms, positive results in a study of functional ischemia, or at the operator’s discretion. Patients were grouped into the revascularization group if at least one non-target lesion was revascularized. Patients who did not receive revascularization were grouped into the non-revascularization group.

### 2.4 Statistical Analysis

The results are presented as either a mean ± standard deviation or a number (percent). The normal distribution of continuous variables was assessed using the Kolmogorov-Smirnov test. For continuous variables, either Student unpaired *t* test or Mann–Whitney U test was used to compare differences between groups. For categorical variables, χ^2^ or Fisher exact test was used. A multivariable Cox proportional hazards model was used to assessed the correlation between clinical variables and rapid progression or revascularization. Variables were included on the basis of their known clinical importance or on the basis of statistical significance with a *p* < 0.15 in univariate comparisons. The variables included age, gender, body mass index, diabetes mellitus, hypertension, ST-segment elevation myocardial infarction (STEMI), family history of CHD, previous myocardial infarction, low-density lipoprotein cholesterol, triglyceride, glycosylated hemoglobin (HbA1c), and American Heart Association/American College of Cardiology (AHA/ACC) lesion classification (B2/C versus A/B1). The hazards ratio (HR) and 95% confidence intervals (95% CI) were used to express the results. Receiver operating characteristic (ROC) curves and area under the ROC curve (AUC) were utilized to evaluate the ability of the selected clinical variables to identify rapid progression or revascularization. All probability values reported were two-tailed, and statistical significance was considered at a *p*-value of less than 0.05. SPSS version 24.0 (IBM Corp., Armonk, NY, USA) was used for calculations.

## 3. Results

### 3.1 Population Characteristics and Clinical Outcomes

**Fig. 1.**
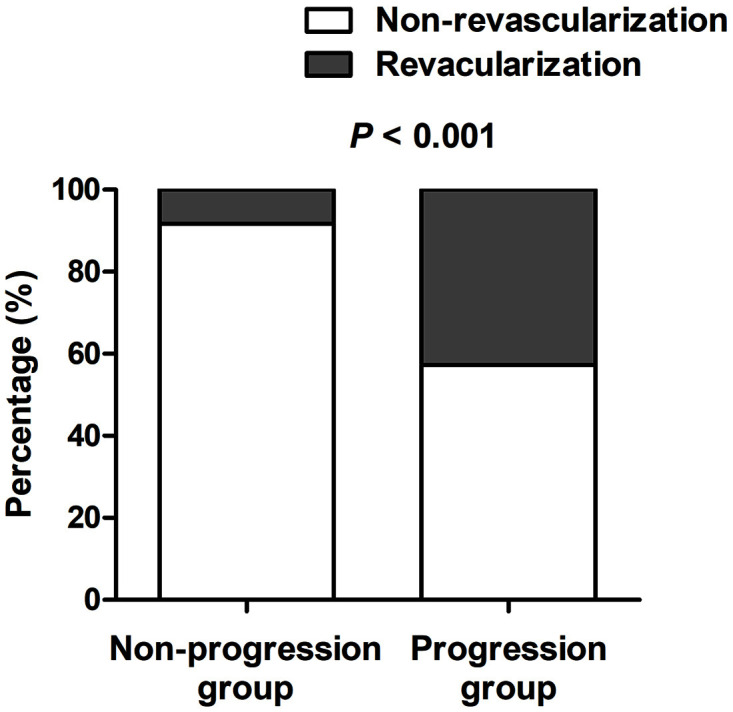
**Incidence of non-target lesion revascularization in progression group and non-progression group**.

In total, 1255 patients who had undergone two serial coronary angiographies were enrolled in the analysis (Table 1). The mean interval between the two CAGs was 14.8 months. 239 patients (19%) had rapid progression of coronary non-target lesions and were grouped into the progression group. The baseline characteristics of the study population in both the progression and non-progression groups are presented in Table 1. Compared with the non-progression group, those in the progression group were younger, had a higher frequency of previous PCI, and elevated triglyceride levels. There were no statistically significant differences found in clinical comorbidities, other biochemical lab values, medications, or the CAG interval between the two groups. 186 patients (14.8%) underwent revascularization of coronary non-target lesions at the time of the second CAG. No significant differences were found in population characteristics between patients who underwent revascularization and those who did not, except for elevated triglyceride levels in the revascularization group (**Supplementary Table 1**). The incidence of non-target lesion revascularization was significantly higher in the progression group than in the non-progression group (42.7% [102 in 239] versus 8.3% [84 in 1016], *p* < 0.001) (Fig. 1 and Table 2). Patients in the progression group also had an increased prevalence of non-target lesion related myocardial infarction and (3.3% versus 1.1%, *p* = 0.010) and all myocardial infarctions (4.6% versus 2.2%, *p* = 0.034) compared with patients in the non-progression group.

**Table 1.** **Baseline characteristics of patients**.

Variables		Non-progression (n = 1016)	Progression (n = 239)	*p*-value
Age (years)		58.3 ± 9.6	56.9 ± 10.1	0.039
Male		817 (80.4)	186 (77.8)	0.360
BMI (${\mathrm{kg/m}}^{2}$)		26.3 ± 3.3	26.6 ± 3.9	0.274
Cigarette use		674 (66.3)	150 (62.8)	0.295
Diabetes mellitus		389 (38.3)	97 (40.6)	0.512
Hypertension		678 (66.7)	154 (64.4)	0.499
Dyslipidemia		674 (66.3)	161 (67.4)	0.762
Peripheral vascular disease		106 (10.4)	17 (7.1)	0.120
STEMI		130 (12.8)	40 (16.7)	0.109
NSTEMI		26 (2.6)	4 (1.7)	0.420
Family history of CHD		78 (7.7)	16 (6.7)	0.604
Previous MI		191 (18.8)	56 (23.4)	0.105
Previous stroke		98 (9.6)	20 (8.4)	0.543
Previous PCI		186 (18.3)	67 (28.0)	0.001
LVEF (%)		62.7 ± 7.2	62.6 ± 6.6	0.614
Biochemistry examinations				
	White blood cell (×${\mathrm{10}}^{9}$/L)	6.9 ± 1.8	7.1 ± 1.9	0.142
	Platelet (×${\mathrm{10}}^{9}$/L)	205.8 ± 52.3	210.3 ± 54.4	0.228
	CRP (mg/L)	5.7 ± 14.2	5.7 ± 9.7	0.995
	ESR (mm/H)	10.4 ± 11.6	11.8 ± 11.7	0.093
	NT-pro BNP (pg/mL)	701.4 ± 461.9	720.8 ± 403.5	0.560
	TC (mmol/L)	4.4 ± 1.1	4.3 ± 1.0	0.485
	LDL-C (mmol/L)	2.6 ± 0.9	2.5 ± 0.8	0.149
	TG (mmol/L)	1.8 ± 1.0	2.1 ± 1.8	0.012
	HbA1c (%)	6.4 ± 1.1	6.5 ± 1.3	0.095
Medications between two CAGs				
	Aspirin	1008 (99.2)	237 (99.0)	0.817
	P2Y12 receptor antagonist	891 (87.7)	210 (87.9)	0.991
	Statin	976 (96.1)	229 (95.8)	0.897
	Interval between two CAGs (month)	14.7 ± 4.5	15.2 ± 4.6	0.100

Data were represented as mean ± standard deviation or n (%). BMI, body mass index; STEMI, ST-segment elevation myocardial infarction; NSTEMI, non-ST-segment elevation myocardial infarction; CHD, coronary heart disease; MI, myocardial infarction; PCI, percutaneous coronary intervention; LVEF, left ventricular ejection fraction; CRP, C-reactive protein; ESR, erythrocyte sedimentation rate; NT-pro BNP, N-terminal pro-B-type natriuretic peptide; TC, total cholesterol; LDL-C, low-density lipoprotein cholesterol; TG, triglyceride; HbA1c, glycosylated hemoglobin; CAGs, coronary angiographies.

**Table 2.** **Clinical outcomes in patients grouped by the presence of rapid progression at second CAG**.

Variables	Non-progression (n = 1016)	Progression (n = 239)	*p*-value
Non-target lesion revascularization	84 (8.3)	102 (42.7)	<0.001
Non-target lesion related myocardial infarction	11 (1.1)	8 (3.3)	0.010
All myocardial infarction	22 (2.2)	11 (4.6)	0.034

Data were represented as n (%). CAG, coronary angiography.

### 3.2 Lesion Characteristics and QCA Analysis of Coronary Non-Target Lesions

In all, 1670 coronary non-target lesions were recorded at the time of the first CAG of which 251 (15.0%) lesions had rapid progression and 1419 (85.0%) showed no progression at the lesion level (Table 3). Lesions with rapid progression were more complex compared to those without progression (frequency of B2+C type lesion was 72.9% versus 61.2% respectively, *p* < 0.001). QCA analysis revealed that lesions with progression had both a smaller reference diameter and a smaller minimum lumen diameter as well as a longer lesion length at the first CAG. The percent diameter stenosis was similar between the groups. However, at the second CAG, minimal lumen diameter was significantly decreased and percent diameter stenosis markedly increased in lesions with rapid progression (both *p* < 0.001). At the second CAG, a total of 194 coronary non-target lesions underwent revascularization (Table 4). These lesions also showed a significantly increased percent diameter stenosis compared with those without revascularization (*p* < 0.001). The incidence of revascularization was significantly lower in lesions without the rapid progression group than those with (6.7% versus 39.4%, *p* < 0.001).

**Table 3.** **Lesion characteristics and QCA analysis of non-target lesions grouped by rapid progression**.

Variables		Non-progression (n = 1419)	Progression (n = 251)	*p*-value
Lesion distribution				0.011
	LM	2 (0.1)	0 (0.0)	
	LAD	511 (36.0)	90 (35.9)	
	LCX	346 (24.4)	81 (32.3)	
	RCA	513 (36.2)	67 (26.7)	
	Dia/OM	47 (3.3)	13 (5.2)	
Lesion location				0.949
	Proximal	622 (43.8)	109 (43.4)	
	Mid	547 (38.5)	100 (39.8)	
	Distal	250 (17.5)	42 (16.7)	
Lesion classification				<0.001
	A+B1	551 (38.8)	68 (27.1)	
	B2+C	868 (61.2)	183 (72.9)	
QCA analysis (first CAG)				
	Reference diameter (mm)	2.9 ± 0.6	2.8 ± 0.6	0.006
	Lesion length (mm)	12.8 ± 7.1	13.8 ± 8.6	0.037
	Minimal lumen diameter (mm)	1.8 ± 0.4	1.7 ± 0.4	<0.001
	Percent diameter stenosis (%)	37.9 ± 9.1	37.8 ± 8.4	0.912
QCA analysis (second CAG)				
	Reference diameter (mm)	2.8 ± 0.6	2.8 ± 0.6	0.022
	Lesions length (mm)	14.3 ± 7.8	17.1 ± 9.8	<0.001
	Minimal lumen diameter (mm)	1.7 ± 0.4	1.1 ± 0.5	<0.001
	Percent diameter stenosis (%)	40.3 ± 10.2	55.9 ± 15.7	<0.001
Revascularization of non-target lesion		95 (6.7)	99 (39.4)	<0.001

Data were represented as mean ± standard deviation or n (%). QCA, quantitative coronary angiography; LM, left main; LAD, left anterior descending artery; LCX, left circumflex artery; RCA, right coronary artery; Dia, diagonal branch; OM, obtuse marginal branch; CAG, coronary angiography.

**Table 4.** **Lesion characteristics and QCA analysis of non-target lesions grouped by revascularization**.

		Non-revascularization (n = 1476)	Revascularization (n = 194)	*p*-value
Lesion distribution				0.179
	LM	2 (0.1)	0 (0.0)	
	LAD	518 (35.1)	83 (42.8)	
	LCX	388 (26.3)	39 (20.1)	
	RCA	513 (34.8)	67 (34.5)	
	Dia/OM	55 (3.7)	5 (2.6)	
Lesion location				0.058
	Proximal	661 (44.8)	70 (36.1)	
	Mid	555 (37.6)	92 (47.4)	
	Distal	260 (17.5)	32 (16.5)	
Lesion classification				0.018
	A+B1	562 (38.1)	57 (29.4)	
	B2+C	914 (61.9)	137 (70.6)	
QCA analysis (first CAG)				
	Reference diameter (mm)	2.8 ± 0.6	2.8 ± 0.5	0.982
	Lesions length (mm)	12.8 ± 7.2	14.2 ± 8.5	0.009
	Minimal lumen diameter (mm)	1.8 ± 0.4	1.7 ± 0.4	0.001
	Percent diameter stenosis (%)	37.7 ± 9.0	39.5 ± 9.0	0.006
QCA analysis (second CAG)				
	Reference diameter (mm)	2.8 ± 0.6	2.8 ± 0.5	0.971
	Lesions length (mm)	14.4 ± 7.8	17.2 ± 10.5	<0.001
	Minimal lumen diameter (mm)	1.7 ± 0.5	1.2 ± 0.5	<0.001
	Percent diameter stenosis (%)	41.2 ± 11.0	54.2 ± 16.6	<0.001

Data were represented as mean ± standard deviation or n (%). The abbreviations as in Table 3.

### 3.3 Association between Clinical Characteristics and Rapid Progression or Revascularization

**Fig. 2.**
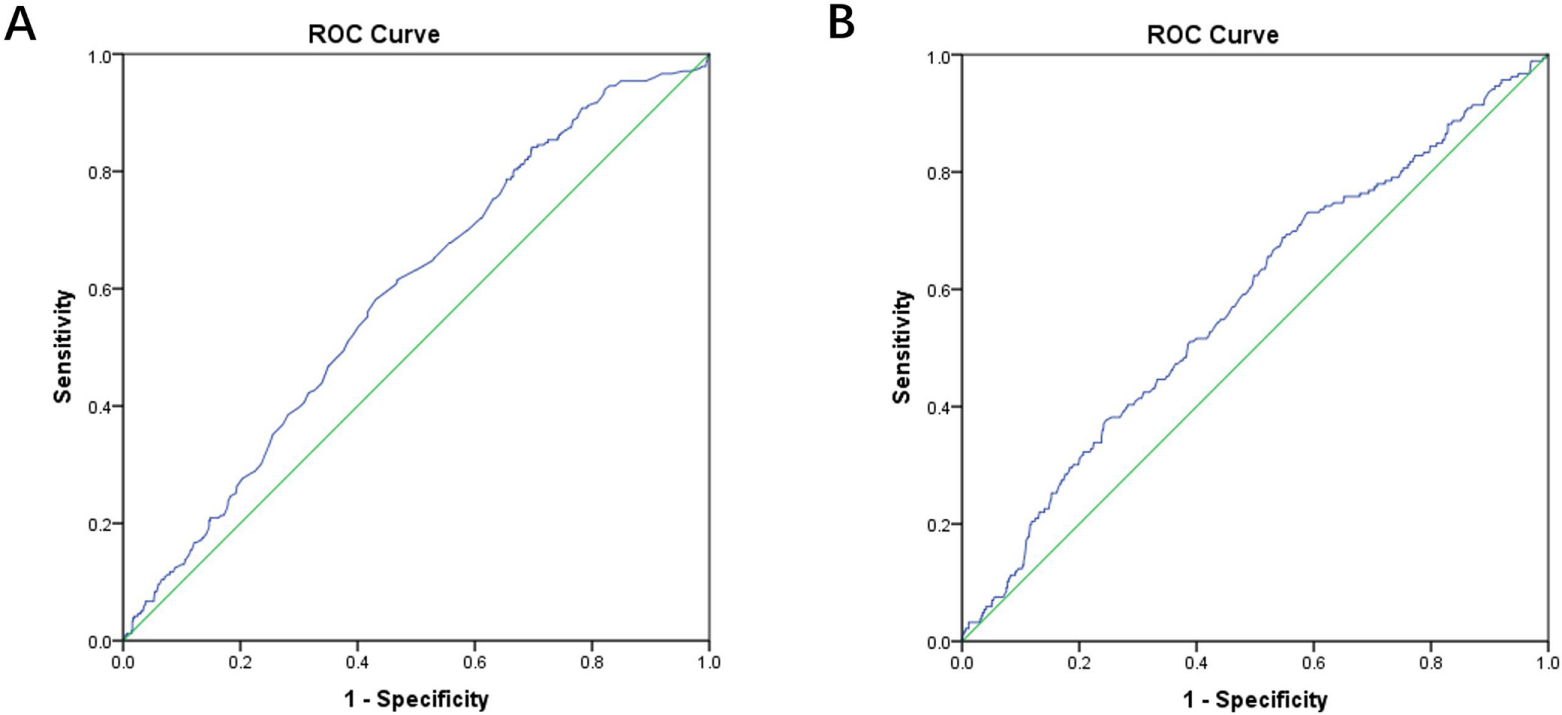
**Receiver-operator characteristic (ROC) curve analysis of clinical characteristics**. (A) ROC curves to identify rapid progression. ROC curves for the clinical factors include hypertension, ST-segment elevation myocardial infarction (STEMI), glycosylated hemoglobin and lesion classification to identify rapid progression. The area under the ROC curve (AUC) was 0.59. (B) ROC curves to identify revascularization. ROC curves for the clinical factors include the level of triglyceride and lesion classification. The AUC was 0.58.

We investigated the association between clinical characteristics and rapid progression in Table 5. Variables such as age, gender, body mass index, hypertension, diabetes mellitus, STEMI, family history of CHD, previous myocardial infarction, low-density lipoprotein cholesterol, triglyceride levels, HbA1c and lesion classification were enrolled into the analysis. In multivariable analyses, hypertension (HR, 0.76; 95% CI, 0.58–1.00; *p* = 0.049), STEMI (HR, 1.46; 95% CI, 1.03–2.07; *p* = 0.035), HbA1c (HR, 1.16; 95% CI, 1.01–1.33; *p* = 0.039) and lesion classification (B2/C versus A/B1) (HR, 1.73; 95% CI, 1.27–2.35; *p* = 0.001) were significant factors associated with rapid progression. The ROC curve for assessing the ability of these factors to identify rapid progression is shown in Fig. 2A. The AUC was 0.59 (95% CI, 0.55–0.63; *p* < 0.001). We then investigated the association between clinical characteristics and revascularization in Table 6. Results showed that the level of triglycerides (HR, 1.10; 95% CI, 1.00–1.20; *p* = 0.040) and lesion classification (B2/C versus A/B1) (HR, 1.53; 95% CI, 1.09–2.14; *p* = 0.014) were significant factors associated with lesion revascularization. The ability of the triglyceride levels and lesion classification to identify lesion revascularization was shown in Fig. 2B with an AUC of 0.58 (95% CI, 0.53–0.62; *p* = 0.001).

**Table 5.** **Association between clinical variables and rapid progression of coronary non-target lesions**.

Item	Variables	Univariable	*p*-value	Multivariable	*p*-value
Non-target lesion progression	Age	0.99 (0.97–1.00)	0.031		
	Male	1.02 (0.75–1.38)	0.914		
	BMI	1.01 (0.98–1.05)	0.469		
	Diabetes mellitus	1.02 (0.78–1.32)	0.906		
	Hypertension	0.79 (0.61–1.03)	0.081	0.76 (0.58–1.00)	0.049
	STEMI	1.53 (1.09–2.15)	0.014	1.46 (1.03–2.07)	0.035
	Family history of CHD	0.88 (0.53–1.46)	0.626		
	Previous MI	1.16 (0.86–1.57)	0.328		
	LDL-C	0.89 (0.77–1.03)	0.108		
	TG	1.10 (1.01–1.19)	0.025		
	HbA1c	1.08 (0.97–1.20)	0.152	1.16 (1.01–1.33)	0.039
	Lesion classification (B2/C versus A/B1)	1.74 (1.29–2.37)	<0.001	1.73 (1.27–2.35)	0.001

Data are presented as hazards ratio (95% confidence interval). Variables listed in the univariable analysis were entered into multivariable analysis. The abbreviations as in Table 1.

**Table 6.** **Association between clinical variables and revascularization of coronary non-target lesions**.

Item	Variables	Univariable	*p*-value	Multivariable	*p*-value
Non-target lesion revascularization	Age	0.99 (0.98–1.01)	0.204		
	Male	0.92 (0.65–1.32)	0.659		
	BMI	1.01 (0.97–1.06)	0.598		
	Diabetes mellitus	0.78 (0.58–1.06)	0.114		
	Hypertension	0.82 (0.61–1.11)	0.205		
	STEMI	1.33 (0.89–1.99)	0.168		
	Family history of CHD	0.84 (0.47–1.50)	0.547		
	Previous MI	0.96 (0.67–1.37)	0.802		
	LDL-C	0.99 (0.84–1.15)	0.851		
	TG	1.10 (1.01–1.20)	0.026	1.10 (1.00–1.20)	0.040
	HbA1c	0.97 (0.85–1.11)	0.688		
	Lesion classification (B2/C versus A/B1)	1.55 (1.11–2.17)	0.010	1.53 (1.09–2.14)	0.014

Data are presented as hazards ratio (95% confidence interval). Variables listed in the univariable analysis were entered into multivariable analysis. The abbreviations as in Table 1.

## 4. Discussion

The objective of this study was to identify the clinical predictors of rapid progression and revascularization of coronary non-target lesions. The results showed that 19.0% of patients experienced rapid progression within a mean interval of 14.8 months and 42.7% of them underwent revascularization. In the multivariate analysis, hypertension, STEMI, HbA1c and lesion classification were significant factors that are associated with rapid progression. In addition, triglyceride levels and lesion classification were essential factors in association with lesion revascularization.

It has been reported that coronary lesions can progress rapidly and lead to adverse cardiac events [7, 14]. In our study, in a mean interval of 14.4 months between two serial CAGs, nearly one in five patients had lesion progression and 42.7% of them underwent revascularization. Patients with rapid progression also had a higher prevalence of myocardial infarction. These results were consistent with previous studies and suggested that rapid progression was quite common and deserves more caution. Previous studies have shown that the majority of acute coronary events were often caused by the progression of mildly stenotic plaques. This progression can be detected through angiographic information that is available for many months to years prior to the event. In the PROSPECT study, Stone and colleagues [15] found that of 106 non-culprit lesions in 697 patients, acute coronary syndrome occurred during a median follow-up period of 3.4 years. And they also found that the mean angiographic diameter stenosis of these progressed lesions was 32% ± 21% at the first CAG. In our study, the progressed lesions had a mean percent diameter stenosis of 37.8% at the first CAG which was also in line with the previous studies. These results suggested that screening clinical risk factors associated with rapid progression has the potential to improve clinical outcomes.

Traditional risk factors for lesion progression are cigarette use and high cholesterol levels. The impact of smoking cessation on the cardiovascular outcomes is still controversial [16]. Our previous work also revealed that smoking cessation was not associated with the reduced frequency of rapid progression [17]. Intensive statin treatment has consistently been shown to reduce total plaque burden and halt progression in multiple imaging studies using different modalities [18]. In our study, there was no difference in cholesterol levels between patients with and without progression at the first CAG and the proportion of patients using statins was also comparable.

Although low-density lipoprotein cholesterol (LDL-C) is a well-known factor in the progression of atherosclerosis, the multivariable analysis in the present study did not show a statistical difference. Possible reasons for the observed results are as follows: (1) The study patients in our hospital have received standard medicine therapy, resulting in low LDL-C levels in both the progression and non-progression populations (2.5 ± 0.8 mmol/L versus 2.6 ± 0.9 mmol/L), indicating a relatively lower effect of LDL-C on progression in this study. (2) The interval between the two CAGs was relatively short, which may have limited the ability to show the contribution of LDL-C levels to rapid progression. (3) Rapid progression or the accelerated atherosclerosis may be initiated by platelet thrombosis, intimal smooth muscle cell proliferation, fibrosis and inflammation rather than lipid deposition. (4) The association between LDL-C and rapid progression in multivariable analysis has statistical but limited clinical value. These results indicate that other factors may involve in the rapid progress of the disease.

In this study, multivariate analysis showed that hypertension, the presentation of STEMI, HbA1c and lesion classification (B2/C versus A/B1) were independent risk factors for rapid progression. The coefficients of these factors are all positive, except that hypertension was negatively associated with rapid progression. Studies from De Luca *et al*. [19] and Yan *et al*. [20] found that patients with CHD and hypertension were more likely to have future cardiovascular events. The negative association between hypertension and rapid progression in our study may have been due to a relatively short CAG interval or simply had statistical significance, but limited clinical importance. Atherosclerosis in non-culprit coronary lesions has previously been shown to be accelerated by the presentation of STEMI [21]. Goldstein *et al*. [22] reported that 39.5% of patients with acute myocardial infarction (AMI) had additional angiographic lesions and that this subgroup of patients had a higher rate of recurrent ischemia. Consistent with previous studies, STEMI was independently associated with rapid progression in our study. Therefore, more attention should be paid to patients presenting with STEMI and additional coronary lesions to prevent future cardiovascular events. We also found that the level of HbA1c was a risk factor for rapid progression. Inaba *et al*. [23] found that accelerated plaque progression was blunted in diabetic patients with an HbA1c level below 6.5%. Similarly, results from Ahmad *et al*. [24] showed greater reductions in minimum luminal diameter in coronary lesions from diabetic patients with a baseline HbA1c level of 6.5% or higher. Our results showed that diabetes mellitus was not a risk factor for rapid progression. These results indicate that glycemic control was more important than the diagnosis of diabetes mellitus in preventing lesion progression.

In addition to demographic features, our work also found that an angiographic feature, AHA/ACC lesion classification, was also a risk factor for rapid progression. In our study, patients with B2/C type lesions were more likely to have rapid lesion progression. Theuerle *et al*. [25] also showed that patients with more complex lesion classifications such as B2 or C type had more major adverse cardiac events following a one-year follow-up. In addition, Qiu *et al*. [26] found that a type C lesion was independently associated with a worse prognosis. We showed that lesion classification was not only a risk factor for rapid progression but also independently associated with future revascularization. Therefore, clinicians should also take the morphological characteristics into consideration when performing a revascularization. Pinilla-Echeverri *et al*. [27] demonstrated a close relationship between lesion angiographic morphology and lesion vulnerability, as assessed by optical coherence tomography. This study suggests that lesion morphology may be a predictor of clinical outcomes, and that patients presenting with these lesions should receive extra attention during routine clinical management. Another risk factor we found for lesion revascularization was the level of triglyceride. High triglyceride levels were associated with an increased risk of CHD [28] and poor clinical outcomes in patients with acute myocardial infarction [29]. In our study, the level of triglycerides was also significantly elevated in the progression compared to the non-progression group. These results suggest that abnormal lipid and glycemic metabolism contribute to lesion progression and eventual revascularization.

There were several limitations to this study. First, the generalizability of our findings was limited by the retrospective nature and being derived from single center, which introduces an element selection bias in the study populations. Second, the evaluation of lesion progression was limited to only coronary angiography. Future studies should consider using combined modalities such as optical coherence tomography and intravascular ultrasound. Third, the results do not apply to all populations and should be the subject of confirmation in larger, prospective studies.

## 5. Conclusions

In conclusion, our study showed that coronary non-target lesions progressed rapidly and undergo revascularization in a short period of time. Risk factors including hypertension, STEMI, HbA1c and AHA/ACC lesion classification were useful to identify patients at high risk for rapid progression. Guideline directed medical therapy should be instituted and more attention should be paid to these patients to prevent future cardiovascular events.

## Data Availability

The datasets generated and/or analyzed during the current study are not publicly available due to restrictions of our hospital but are available from the corresponding author on reasonable request.

## Abbreviations

BMI, body mass index; STEMI, ST-segment elevation myocardial infarction; NSTEMI, non-ST-segment elevation myocardial infarction; CHD, coronary heart disease; MI, myocardial infarction; PCI, percutaneous coronary intervention; LVEF, left ventricular ejection fraction; CRP, C-reactive protein; ESR, erythrocyte sedimentation rate; NT-pro BNP, N-terminal pro-B-type natriuretic peptide; TC, total cholesterol; LDL-C, low-density lipoprotein cholesterol; TG, triglyceride; HbA1c, glycosylated hemoglobin; CAG, coronary angiography; QCA, quantitative coronary angiograph; LM, left main; LAD, left anterior descending artery; LCX, left circumflex artery; RCA, right coronary artery; Dia, diagonal branch; OM, obtuse marginal branch.

## Supplementary Material

Supplementary material associated with this article can be found, in the online version, at https://doi.org/10.31083/j.rcm2507251.
