# Supplementary material for: Clinical Predictors of the Rapid Progression and Revascularization of Coronary Non-Target Lesions: A Serial Angiographic Study
Source: Rev Cardiovasc Med. 2024 Jul 8;25(7):251. doi: 10.31083/j.rcm2507251 (PMC11317350; doi:10.31083/j.rcm2507251)
Supplement: Supplementary file 1 [file 2153-8174-25-7-251-s1.docx]

**Supplementary Table 1. Baseline characteristics of patients with coronary heart disease grouped by the presence of revascularization of coronary non-target lesion.**

| Variables | Non-revascularization  (n=1069) | Revascularization  (n=186) | P-value |
| --- | --- | --- | --- |
| Age (years) | 58.2 ± 9.8 | 57.3 ± 9.0 | 0.265 |
| Male | 855 (80.0) | 148 (79.6) | 0.897 |
| BMI (kg/m^2^) | 26.3 ± 3.2 | 26.5 ± 4.1 | 0.415 |
| Cigarette use | 707 (66.1) | 117 (62.9) | 0.391 |
| Diabetes mellitus | 422 (39.5) | 64 (34.4) | 0.190 |
| Hypertension | 711 (66.5) | 121 (65.1) | 0.698 |
| Dyslipidemia | 715 (66.9) | 120 (64.5) | 0.527 |
| Peripheral vascular disease | 110 (10.3) | 13 (7.0) | 0.162 |
| STEMI | 142 (13.3) | 28 (15.1) | 0.515 |
| NSTEMI | 29 (2.7) | 1 (0.5) | 0.073 |
| Family history of CHD | 82 (7.7) | 12 (6.5) | 0.560 |
| Previous MI | 210 (19.6) | 37 (19.9) | 0.937 |
| Previous stroke | 103 (9.6) | 15 (8.1) | 0.498 |
| Previous PCI | 217 (20.3) | 36 (19.4) | 0.767 |
| LVEF (%) | 62.7 ± 7.1 | 62.4 ± 6.8 | 0.535 |
| Biochemistry examinations |  |  |  |
| White blood cell (×10^9^/L) | 6.9 ± 1.8 | 6.8 ± 1.8 | 0.643 |
| Platelet (×10^9^/L) | 206.7 ± 53.0 | 206.3 ± 51.4 | 0.919 |
| CRP (mg/L) | 5.7 ± 14.2 | 5.4 ± 7.9 | 0.729 |
| ESR (mm/H) | 10.5 ± 11.5 | 11.5 ± 12.1 | 0.300 |
| NT-pro BNP (pg/ml) | 700.9 ± 452.8 | 728.3 ± 443.6 | 0.452 |
| TC (mmol/L) | 4.3 ± 1.1 | 4.5 ± 1.0 | 0.179 |
| LDL-C (mmol/L) | 2.6 ± 0.9 | 2.6 ± 0.8 | 0.904 |
| TG (mmol/L) | 1.9 ± 1.1 | 2.1 ± 1.8 | 0.019 |
| HbA1c (%) | 6.4 ± 1.1 | 6.4 ± 1.2 | 0.701 |
| Medications between two CAGs |  |  |  |
| Aspirin | 1061 (99.3) | 184 (98.9) | 0.501 |
| P2Y12 receptor antagonist | 941 (88.0) | 160 (86.0) | 0.206 |
| Statin | 1026 (96.0) | 179 (96.2) | 0.728 |
| Interval between two CAGs (month) | 14.8 ± 4.4 | 14.7 ± 4.8 | 0.820 |

Data were represented as mean ± standard deviation or n (%).

BMI: body mass index; STEMI: ST-segment elevation myocardial infarction; NSTEMI: non-ST-segment elevation myocardial infarction; CHD: coronary heart disease; MI: myocardial infarction; PCI: percutaneous coronary intervention; LVEF: left ventricular ejection fraction; CRP: C-reactive protein; ESR: erythrocyte sedimentation rate; NT-pro BNP: N-terminal pro-B-type natriuretic peptide TC: total cholesterol; LDL-C: low-density lipoprotein cholesterol; TG: triglyceride; HbA1c: glycosylated hemoglobin; CAG: coronary angiography.
